# Cervical Cancer in Mexico: From a Renowned Vaccination Program to Unfulfilled Needs in Treatment Access

**DOI:** 10.7759/cureus.61553

**Published:** 2024-06-02

**Authors:** Irving E Sanchez-Rodriguez, Yaritza G Medina-Gomez, Ricardo I Balderrama-Ibarra, Jan C Ramos-Vega, Kiven W Ramos-Vega

**Affiliations:** 1 Radiation Oncology, Mexican Institute of Social Security (IMSS), Guadalajara, MEX; 2 Medical Oncology, Mexican Institute of Social Security (IMSS), Puerto Vallarta, MEX; 3 Radiation Oncology, Mexican Institute of Social Security (IMSS), Ciudad Obregon, MEX; 4 School of Medicine, Universidad Autónoma de Guadalajara, Zapopan, MEX

**Keywords:** hpv vaccination program, human papillomavirus (hpv), gynaecologic oncology, radiation oncoolgy, uterine cervical cancer, healthcare systems around the world, global healthcare systems

## Abstract

Mexico's national human papillomavirus (HPV) vaccination program was established in 2008, providing free access to HPV vaccines and quickly becoming an immense success story, achieving significant coverage among young Mexican females. However, despite these efforts and notable achievements, cervical cancer caused mainly by HPV remains a challenging issue among Mexican women aged 15 years or older. A critical obstacle faced by women in the country is a lack of early detection and screening resources, coupled with delays in diagnosis and treatment, exacerbated by the poor distribution of already insufficient healthcare resources. This situation creates adverse conditions for the female demographic in the country. Our editorial aims to draw attention to the urgent need to improve access to adequate prevention, screening, and treatment for cervical cancer patients in Mexico, advocating for a collective effort between the Mexican government, public health professionals, and civil society.

## Editorial

According to statistics from the Global Cancer Observatory (formerly GLOBOCAN) in 2022, Mexico holds a distressing second position in both incidence (10,348 cases) and mortality (4,909 cases) among Mexican women, within an estimated population of 49.6 million females aged 15 years or older [1]. This contrasts with worldwide data, where cervical cancer ranks as the fourth most common cancer, underscoring the urgent need to reinforce efforts in screening, and treatment access.

Mexico implemented its national human papillomavirus (HPV) vaccination initiative in 2008, offering free access to HPV vaccines and rapidly emerging as a resounding success, particularly in reaching and vaccinating a substantial portion of young Mexican females. By 2022, Mexico's national HPV vaccination program had achieved over 90% vaccine coverage among young females under 15 years old [2]. Nonetheless, despite these endeavors and notable accomplishments, cervical cancer, primarily attributable to HPV, persists as a significant challenge. 

Since its inception in 1974, the national cervical cancer screening and early detection program faced significant challenges, resulting in an approximate mortality rate of 80,000 cases between 1980 and 1999. The main issues include limited access to adequate screening methods, early detection practices, and treatment, disproportionately affecting women with low socioeconomic status. Notably, only 30% of women in rural areas of Mexico get screened with cervical cytology, compared to 64% in suburban areas [3].

Moreover, there has been a lack of clear intentions from public health authorities to address this situation, particularly in rural areas where access to services is even more challenging. This issue is not exclusive to cervical cancer caused by HPV; however, due to its known disease presentation and relatively preventable course, it should be given high priority in terms of intervention.

In 2020, the World Health Organization (WHO) implemented a strategy to eliminate cervical cancer to reduce the incidence rate to just four cases per 100,000 women in all countries by 2030 [4]. The current incidence in Mexico is 5.8 cases per 100,000 inhabitants.

To achieve the goal set by WHO, three main objectives were established: (i) Prevention, specifically aiming for 90% coverage of HPV vaccination among girls under 15 years old, which has been achieved; (ii) Early detection, targeting 70% of women aged 35-45 years for proper molecular screening. Mexico currently faces disparities in access to adequate screening methods, especially in rural areas; (iii) Guaranteed treatment for 90% of women diagnosed with cervical cancer. As per National regulations, all women must be referred to a specialized cancer care unit once the diagnosis of cervical cancer has been established. Although referrals are promptly done, patients still experience very long delays in receiving adequate cancer care due to the disproportional demand.

The lack of resources directed towards oncological care, such as radiotherapy, particularly in locally advanced cancer where radiotherapy plays a central role (including external beam radiotherapy and brachytherapy), combined with an overwhelmed public health system, significantly limits the quality of care that affected women receive. This is evident in poor screening and limited treatment access, overshadowing the success achieved by the national HPV vaccination program in previous years and resulting in higher-than-expected mortality rates.

According to our most recent national data about radiotherapy availability and access, Mexico has 103 radiotherapy centers, accounting for a total of 162 radiotherapy machines (both linear accelerators and cobalt units), which translates to 1.32 radiotherapy machines per million inhabitants. Additionally, only 66 brachytherapy units are available, which are essential for the management of cervical cancer, especially in locally advanced stages. This figure falls far below international recommendations. The International Atomic Energy Agency suggests a goal of at least four treatment facilities per million inhabitants, while the Organization for Economic Co-operation and Development recommends an average range of 2-3.5 treatment machines per million in low and middle-income countries [5]. These statistics shed light on the treatment access issues, particularly the delays experienced by women with locally advanced tumors requiring a multidisciplinary approach, primarily driven by radiotherapy.

To summarize, while Mexico's national HPV vaccination program has seen immense success, it only marks the start of a long and challenging fight against a persistent disease. This disease continues to yield high mortality rates and disrupt the quality of life for a significant number of Mexican women. Cervical cancer, caused by HPV, requires urgent intervention. Expanding treatment options and facilitating accessible screening are crucial to properly addressing the overwhelming data regarding incidence and mortality. Coordinated efforts between the Mexican government, public health officials, and civil organizations are imperative to start overcoming this serious issue. No woman should die from a preventable and treatable disease.
